# Antioxidant Potential of Pine Needles: A Systematic Study on the Essential Oils and Extracts of 46 Species of the Genus *Pinus* †

**DOI:** 10.3390/foods10010142

**Published:** 2021-01-12

**Authors:** Aikaterini Koutsaviti, Samer Toutoungy, Rouba Saliba, Sofia Loupassaki, Olga Tzakou, Vassilios Roussis, Efstathia Ioannou

**Affiliations:** 1 Section of Pharmacognosy and Chemistry of Natural Products, Department of Pharmacy, National and Kapodistrian University of Athens, Panepistimiopolis Zografou, 15771 Athens, Greece; kkoutsaviti@pharm.uoa.gr (A.K.); tzakou@pharm.uoa.gr (O.T.); roussis@pharm.uoa.gr (V.R.); 2 Department of Food Quality and Chemistry of Natural Products, Mediterranean Agronomic Institute of Chania—Centre International de Hautes Etudes Agronomiques Méditerranéennes, 73100 Chania, Greece; tutunji@hotmail.com (S.T.); rouba.saliba@hotmail.com (R.S.); sofia@maich.gr (S.L.)

**Keywords:** *Pinus*, pine needles, antioxidant activity, chemiluminescence, secondary metabolites, chromatographic separations

## Abstract

The antioxidant activity of the essential oils, as well as of the organic and hydroethanolic extracts, of the fresh needles of 54 pine taxa was evaluated using the peroxy-oxalate and luminol chemiluminescence assays. Among all evaluated essential oils, *P. canariensis* and *P. attenuata* displayed the highest levels of activity. *P. contorta* var. *murrayana*, followed by *P. nigra* var. *caramanica*, exhibited the highest antioxidant capacity among the organic extracts, while the most active hydroethanolic extract was that of *P. nigra* subsp. *nigra*. Based on the overall levels of activity, the latter taxon was selected for phytochemical analysis targeting the isolation of the bioactive constituents. As such, the organic extract of *P. nigra* subsp. *nigra* was subjected to chromatographic separations to yield 11 secondary metabolites (**1−11**) that were evaluated for their antioxidant activity. Nonetheless, the isolated compounds were found to be less active than the crude extract, thus suggesting the potential role of synergism.

## 1. Introduction

In recent years, interest towards finding new antioxidant agents derived from natural sources has increased, since synthetic antioxidant compounds currently in use, such as butylated hydroxy-anisole (BHA) and tertiary butyl hydroquinone (TBHQ), may induce serious side effects (e.g., carcinogenesis) [1,2]. Apart from preventing food deterioration by militating against the activity of reactive oxygen species, natural antioxidant agents may also add nutritional value as functional food ingredients [3].

Pines are monoecious woody plants, mostly seen as tall trees and rarely as shrubs, with distinctive needle-shaped evergreen leaves, encountered in a variety of terrestrial environments and climatic zones in both hemispheres, mainly distributed over the northern hemisphere, while they also occur in subtropical and tropical areas of Central America and Asia [4,5]. The genus *Pinus*, including approximately 110 species [4,5], is important from an ecological point of view, since its representatives form extended forests either in pure stands or mixed with other conifers. Furthermore, from an economic point of view, pines are a valuable source of nuts and seeds, as well as resin, but also of pulp and paper, timber and construction materials.

The genus *Pinus* is a well-known source of antioxidants, mainly phenolic compounds, including procyanidins and other flavonoids and phenolic acids, already available in the market as food supplements or phytochemical remedies, such as Pycnogenol^™^, a standardized bark extract from *Pinus maritima*, with a remarkable array of biological activities, used also in the treatment of chronic inflammation and circulatory dysfunction [6]. In the last 25 years, various pine extracts and preparations have exhibited significant health-promoting activities, e.g., protective activity against alcohol-induced liver disease or against lipopolysaccharide-induced inflammation, hippocampal memory-enhancing activity, and activity for the early management of dyslipidemia, that can be potentially useful in food, functional food, and food supplement industries [7,8,9,10,11,12,13,14,15].

Besides the traditional use of pine seeds for human consumption either as edible raw nuts or in cooked dishes due to their high nutritional value and enticing taste [7], the use of pines cones, needles, bark and oil as food or food ingredients has already been established and accepted in the EU [16]. Pine needles have been used as herb tea in Estonian folk medicine [17], while pine needle-based food products, such as pine needle powder, wine and herbal teas, have become quite popular in the Korean food market [18]. It is worth noting that in recent years, the use of pine needles in herbal teas and as an ingredient in various food recipes has gained significant interest [19,20].

The aim of the present study was the investigation of the antioxidant potential of the essential oils and extracts of fresh needles from 46 pine species, including 37 and 17 taxa of the subgenera *Pinus* and *Strobus*, respectively, using two methods based on the measurement of chemiluminescence (CL), with the prospect of finding new natural antioxidant agents for the nutraceutical, food and food supplement industries, thus capitalizing on the renewable pine needle biomass as a sustainable and eco-friendly approach.

## 2. Materials and Methods

### 2.1. General Experimental Procedures

NMR spectra were recorded on Bruker AC 200 and Bruker DRX 400 spectrometers (Bruker BioSpin GmbH, Rheinstetten, Germany). Chemical shifts are given on a *δ* (ppm) scale using TMS as internal standard. The 2D NMR experiments (HSQC, HMBC, COSY, NOESY) were performed using standard Bruker pulse sequences. Optical rotations were measured on a Perkin Elmer model 341 polarimeter (PerkinElmer Instruments, Norwalk, CT, USA) with a 1 dm cell. UV spectra were obtained on a Shimadzu UV-160A spectrophotometer (Shimadzu Europa GmbH, Duisburg, Germany). IR spectra were obtained on a Bruker Tensor 27 spectrometer (Bruker Optik GmbH, Ettlingen, Germany). High-resolution ESI mass spectra were measured on a Thermo Scientific LTQ Orbitrap Velos mass spectrometer (Thermo Fisher Scientific, Bremen, Germany). Low-resolution EI and CI mass spectra were measured on a Thermo Electron Corporation DSQ mass spectrometer a Direct-Exposure Probe (Thermo Fisher Scientific, Bremen, Germany), using CH_4_ as reagent gas. Normal- and reversed-phase column chromatography separations were performed with Kieselgel Si 60 (Merck, Darmstadt, Germany) and Kieselgel RP-18 (Merck, Darmstadt, Germany), respectively. HPLC separations were conducted on an Agilent 1100 liquid chromatography system equipped with a refractive index detector (Agilent Technologies, Waldbronn, Germany) or a CECIL 1100 Series liquid chromatography pump (Cecil Instruments Ltd., Cambridge, UK) equipped with a GBC LC-1240 refractive index detector (GBC Scientific Equipment, Braeside, VIC, Australia), using the following columns: Kromasil 100 SIL 5 μm (MZ-Analysentechnik GmbH, Mainz, Germany, 250 mm × 8 mm i.d.), Econosphere 100 SIL 10u (Grace, Columbia, MD, USA, 250 mm × 10 mm i.d.), Νucleosil SIL 50-7 (Macherey-Nagel GmbH & Co. KG, Düren, Germany, 250 mm × 10 mm i.d.), Kromasil 100 C_18_ 5 μm (MZ-Analysentechnik GmbH, Mainz, Germany, 250 mm × 8 mm i.d.) or Econosphere C_18_ 10u (Grace, Columbia, MD, USA, 250 mm × 10 mm i.d.). Thin layer chromatography (TLC) was performed with Kieselgel 60 F_254_ aluminum plates (Merck, Darmstadt, Germany) and spots were detected after spraying with H_2_SO_4_ in MeOH (15% *v*/*v*) reagent and heating at 100 °C for 1 min. All solvents used for preparative and analytical purposes (CH_2_Cl_2_, MeOH, cHex, EtOAc, EtOH, MeCN) were of analytical or HPLC grade from LAB-SCAN Analytical Sciences (Gliwice, Poland), whereas CDCl_3_ and CD_3_OD used for NMR spectroscopic analyses were from Deutero GmbH (Kastellaun, Germany). Hydrogen peroxide (H_2_O_2_, 35%), *β*-carotene, quercetin and CoCl_2_^.^6H_2_O were from Merck (Darmstadt, Germany), while 9,10-diphenylanthracene (9,10-DPA), bis(2,3,6-trichlorophenyl) oxalate (TCPO), ethylenediaminetetraacetic acid (EDTA), imidazole, luminol, NaOH and H_3_BO_3_ were from Sigma-Aldrich (St. Louis, MI, USA).

### 2.2. Plant Material

Fresh needles of 54 taxa of genus *Pinus*, namely 37 taxa of subgenus *Pinus* and 17 taxa of subgenus *Strobus*, were collected from either well-documented wild localities or from botanical gardens, as previously described [21]. Voucher specimens of the taxa have been deposited at the Herbarium of the Section of Pharmacognosy and Chemistry of Natural Products, Department of Pharmacy, National and Kapodistrian University of Athens. The moisture content of the fresh needles ranged between 49–57%, as determined after incubation in an oven at 80 °C for 6 h.

### 2.3. Isolation of Essential Oils

Fresh needles of each sample (30–50 g) were cut into small pieces (0.5–1 cm) and separately subjected to hydro-distillation for 3 h using a modified Clevenger-type apparatus with a water-cooled receiver, in order to reduce overheating artifacts. The isolated essential oils were taken up in pentane, dried over anhydrous sodium sulfate and stored at 4 °C until analyzed.

### 2.4. Preparation of Extracts

Fresh needles of each sample were cut into small pieces (0.5–1 cm) and divided into two parts (A and B), of approx. 0.5 g each. Part A was macerated with 5 mL CH_2_Cl_2_/EtOH (2:1) to prepare the organic extract, while part B was macerated with 5 mL EtOH/H_2_O (1:2) to prepare the hydroethanolic extract. In both cases, extraction was repeated twice for 24 h each time at 25 °C. After evaporation of the solvents in vacuo, the extracts were weighted and stored at 4 °C until assayed.

### 2.5. Extraction and Isolation of Secondary Metabolites from P. nigra subsp. nigra

Fresh needles of *P. nigra* subsp. *nigra* (60.0 g) were exhaustively extracted with CH_2_Cl_2_/EtOH (2:1) (three times with fresh volume of solvents; no additional amount of residue was obtained afterwards) at 25 °C. After evaporation of the solvents in vacuo, the crude extract (1.4 g) was subjected to gravity column chromatography on silica gel, using cHex with increasing amounts of EtOAc, followed by EtOAc with increasing amounts of MeOH as the mobile phase, to afford 16 fractions (1–16). Fractions 3 (28.8 mg), 4 (47.6 mg), 5 (24.9 mg) and 6 (49.5 mg) were separately purified by normal-phase HPLC, using cHex/EtOAc (85:15) as eluent, to yield **1** (9.2 mg), **2** (0.5 mg), **7** (3.2 mg), **8** (6.8 mg), **9** (15.2 mg) and *β*-sitosterol (4.7 mg). Fractions 7 (60.8 mg) and 8 (34.7 mg) were separately purified by normal-phase HPLC, using cHex/EtOAc (75:25) as eluent, to yield **3** (3.0 mg), **4** (6.9 mg), **6** (11.2 mg), **9** (6.8 mg) and **10** (0.7 mg). Fraction 10 (22.2 mg) was purified by normal-phase HPLC, using cHex/EtOAc (70:30) as eluent, to yield **5** (4.7 mg). Fraction 14 (40.1 mg) was subjected to reversed-phase HPLC, using MeOH/H_2_O (50:50) as eluent, to yield **11** (3.5 mg).

*5,4′-Dihydroxy-3,6,7-trimethoxy-8-C-methylflavone* (**10**): Yellow solid; ^1^H NMR (CD_3_OD, 400 MHz) *δ* 12.47 (s, 1H, 5-OH), 8.11 (dd, *J* = 8.8, 2.1, 2H, H-2′ and H-6′), 6.97 (dd, *J* = 8.8, 2.1, 2H, H-3′ and H-5′), 3.99 (s, 3H, 7-OMe), 3.92 (s, 3H, 6-OMe), 3.86 (s, 3H, 3-OMe), 2.15 (s, 3H, 8-Me); ^13^C NMR (CD_3_OD, 50.3 MHz) *δ* 157.8 (C-4′), 157.2 (C-7), 155.4 (C-2), 153.9 (C-8a), 138.9 (C-3), 132.6 (C-6), 130.4 (C-2′ and C-6′), 123.3 (C-1′), 115.6 (C-3′ and C-5′), 113.4 (C-8), 61.7 (6-OMe), 60.9 (7-OMe), 60.0 (3-OMe), 7.9 (8-Me).

### 2.6. Evaluation of Antioxidant Activity Using the Peroxy-Oxalate Chemiluminescence Assay

The antioxidant activity of the essential oils was evaluated using the peroxy-oxalate chemiluminescence (POCL) assay, based on the measurement of CL as a result of the oxidation of an aryl oxalate ester by H_2_O_2_ in the presence of 9,10-DPA as a fluorophore (activator) and developed for assessing the hydrogen peroxide scavenging activity of low polarity hydrophobic samples [22]. Briefly, 0.2 mL of TCPO solution (0.45 mM) and 0.05 mL of the sample solution (at least three different concentrations were tested) or solvent (EtOAc) in the case of blank measurements were placed in a cuvette and immediately 1.8 mL 9,10-DPA solution (0.5 mM), 0.2 mL imidazole solution (4.5 mM) and 0.025 mL H_2_O_2_ solution (2.25 mM) were added and mixed well for 5 s. All solutions were prepared in EtOAc/MeCN (9:1), with the exception of the sample solutions which were prepared in EtOAc. CL was continuously monitored in a JENWAY 6200 fluorimeter (Jenway Ltd., Essex, UK), keeping the lamp off and using only the photomultiplier of the apparatus, until the reaction reached a plateau and CL intensity was recorded.

### 2.7. Evaluation of Antioxidant Activity Using the Luminol Chemiluminescence Assay

The antioxidant activity of the organic and hydroethanolic extracts, as well as of the isolated metabolites was evaluated using the luminol chemiluminescence (LCL) assay, based on the measurement of CL as a result of the oxidation of luminol by H_2_O_2_ in the presence of cobalt (II) as a transition metal and EDTA as a metal chelator, and developed for assessing the hydroxyl free radical scavenging activity of medium and high polarity samples [23,24]. Briefly, 1 mL of Co(II)/EDTA solution (8.4 mM CoCl_2_^.^6H_2_O and 34.25 mM EDTA) and 0.1 mL of luminol solution (0.56 mM) were placed in a cuvette and mixed well for 15 s and subsequently 0.025 mL H_2_O_2_ solution (5.4 mM) and 0.025 mL of the sample solution (at least three different concentrations were tested) or solvent (MeOH) in the case of blank measurements were added and mixed well for 15 s. The Co(II)/EDTA and luminol solutions were prepared in borate buffer (H_3_BO_3_ 0.05 M, adjusted to pH 9 using NaOH 1 M), the H_2_O_2_ solution was prepared in H_2_O and the sample solutions were prepared in MeOH. CL was continuously monitored in a LS-55 fluorescence spectrometer (PerkinElmer, Inc., Waltham, MA, USA), until the reaction reached a plateau and CL intensity was recorded.

### 2.8. Determination of Antioxidant Activity and Statistical Analysis

For both assays, an equation in the form I_0_/I = a × C ± b was obtained by plotting I_0_/I against C, where I_0_ is the initial CL intensity recorded for the blank, I is the reduced CL intensity recorded after the addition of the sample and C is the concentration of the sample (in μg/mL). Correlations were established using linear regression analysis (with a coefficient *R*^2^ > 0.98), employing Microsoft Office Excel 2007 software. Assignments a and b represent the gradient and the intercept of the equation, respectively. The concentration necessary to decrease the CL intensity by 50% (IC_50_) was calculated by setting I_0_/I = 2. All measurements were performed at least in three independent experiments and data are presented as mean ± SEM (standard error of the mean).

## 3. Results and Discussion

### 3.1. Evaluation of the Antioxidant Activity of Essential Oils

**Figure 1:**
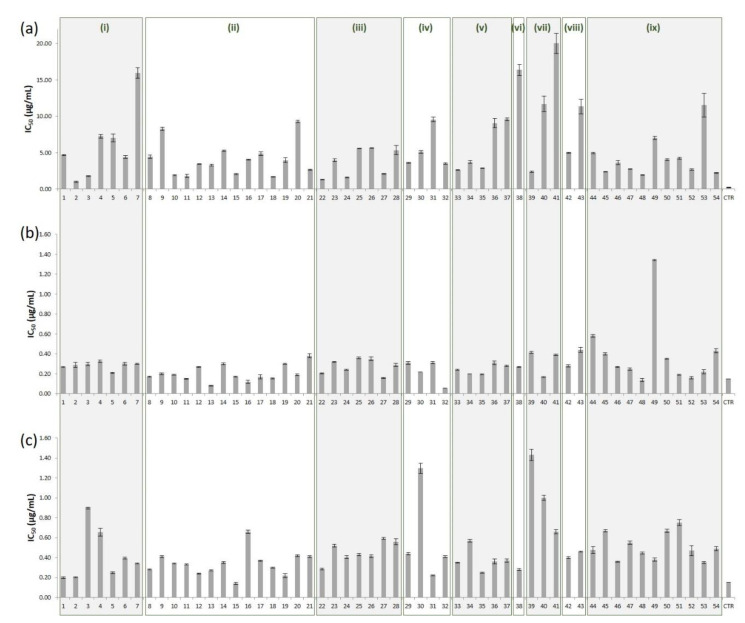
Graphical representation of the antioxidant activity (expressed as IC_50_ in μg/mL) exerted by (**a**) the essential oils, (**b**) the organic (CH_2_Cl_2_/EtOH 2:1) and (**c**) the hydroethanolic (EtOH/H_2_O 1:2) extracts of the fresh needles of 54 *Pinus* taxa, in comparison to that of the positive control (CTR: *β*-carotene for (**a**) and quercetin for (**b**) and (**c**)). The various boxes represent the following subsections: (i) *Pinaster*, (ii) *Pinus*, (iii) *Australes*, (iv) *Contortae*, (v) *Ponderosae*, (vi) *Balfourianae*, (vii) *Cembroides*, (viii) *Gerardianae*, (ix) *Strobus*.

The antioxidant activity of the essential oils obtained from the fresh needles of 46 pine species, including 37 and 17 taxa of the subgenera *Pinus* and *Strobus*, respectively, was evaluated using the POCL assay. According to the results of the evaluation (Table 1, Figure 1a), the IC_50_ values of the pine needle essential oils ranged from 1.00 ± 0.08 (*P. canariensis*) to 20.03 ± 2.77 (*P. cembroides* var. *monophylla*). Besides *P. canariensis* oil which exhibited the most significant antioxidant activity, high levels of activity were also displayed by the essential oils of *P. attenuata* (1.30 ± 0.02), *P. muricata* (1.60 ± 0.09), *P. sylvestris* var. *scotica* (1.67 ± 0.05), *P. halepensis* (1.78 ± 0.17), *P. mugo* var. *prostrata* (1.79 ± 0.21), *P*. *mugo* (1.89 ± 0.16) and *P. monticola* (1.94 ± 0.09). As can be observed, with the exception of the latter needle oil derived from a species belonging to the subgenus *Strobus*, the most active essential oils were obtained from taxa of the subgenus *Pinus*.

Analysis of the chemical composition of the essential oils evaluated for their antioxidant activity in the present study has shown that mono- and sesquiterpene derivatives characterize the majority of the essential oils [21]. In most cases, α- and β-pinene were the major representatives of the monoterpene fraction. However, occasionally β-phellandrene and/or δ-3-carene were also present in high percentages. The sesquiterpene group was characterized by germacrene D, while the levels of diterpenes varied notably. Germacrene D was one of the common main metabolites among the three most active samples (*P. canariensis* 44.0%*, P. attenuata* 29.0%, and *P. muricata* 41.5%), and while it was detected in notably lower amounts in the essential oils of the following in activity order *P. sylvestris* var. *scotica* (5.1%) and *P. mugo* var. *prostrata* (2.8%), its oxygenated derivative germacrene D-4-ol reached a relatively higher percentage (10.0% and 6.0%, respectively). Instead, the major metabolite in *P. halepensis* needle oil was β-caryophyllene (19.0%). It should be noted though that no clear pattern correlating the antioxidant effect and the chemical composition of the investigated essential oils can be observed overall. Thus, according to our results and in agreement with the literature data [25], it can be deduced that the antioxidant activity exhibited by our samples may be a result of synergism, since pinenes, ubiquitous constituents of pine essential oils often appearing as major components, do not possess antioxidant properties [26]. On the other hand, terpene derivatives such as germacrene D, β-caryophyllene, and γ-terpinene have been reported to exert antioxidant activity [27].

The antioxidant activity of the Aleppo pine (*P. halepensis*) needle oils from Algeria was studied using four different assays, namely 2,2-diphenyl-1-picrylhydrazyl radical scavenging (DPPH), *β*-carotene bleaching (BCB), iron (II) chelating ability employing the Fe^2+^-ferrozine system (FICA) and potassium ferricyanide reducing power (PFRAP) assays, and high levels of activity, especially for a specific chemotype rich in caryophyllene oxide, were also observed, as in our case [28,29]. The high antioxidant potential of *P. halepensis* essential oil was further verified by Postu et al. who observed remarkable activity in the DPPH and 2,2-azino-bis-(3-ethylbenzothiazoline-6-sulphonate) radical cation scavenging (or Trolox equivalent antioxidant capacity, ABTS/TEAC) assays [30]. The antioxidant potential of *P. mugo* essential oil has been evaluated by Grassmann et al., employing a variety of biochemical tests in both aqueous (e.g., Fenton system, xanthine oxidase-induced superoxide radical formation) and more lipophilic environments (e.g., ACC-cleavage by activated neutrophils in whole blood, copper-induced oxidation of low-density lipoprotein), observing good antioxidant activity in more lipophilic rather than in aqueous environments [31]. In contrast, Kurti et al. observed a rather low to moderate DPPH radical scavenging activity for the needle essential oil of *P. mugo* from Kosovo [32]. High to moderate activity, as observed in the present study, for the needle oil of the Himalayan blue pine (*P. wallichiana*), was also noted by Dar et al. using the DPPH assay [26]. On the other hand, the essential oil of the Swiss stone pine (*P. cembra*), which showed high to moderate activity in our study, has previously exhibited rather weak DPPH radical scavenging activity [33]. The needle essential oil of the Japanese black pine (*P. thunbergii*) exerted a strong DPPH radical scavenging potential, as in our study, but insignificant nitrite radical scavenging activity [34]. *P. tabuliformis*, which exhibited moderate levels of activity in the present study, has also previously displayed moderate antioxidant activity when evaluated using the DPPH, ABTS/TEAC and ferric reducing antioxidant power (FRAP) assays [35]. In the study of Yener et al., the essential oil of *P. brutia* exhibited strong iron (II) chelating ability and relatively lower levels of activity in the DPPH and PFRAP assays, whereas the foliage essential oil of umbrella pine (*P. pinea*) displayed weak iron (II) chelating ability, as well as weak reducing power [36]. Moreover, in the study of Ustun et al., the essential oil of *P. brutia* exhibited weak activity in the PFRAP assay, while *P. sylvestris* essential oil showed moderate iron (II) chelating ability [37]. *P. sylvestris* essential oil and its fractions from Kosovo were also tested as DPPH radical scavenging agents, displaying a weak to moderate potential [32]. In the same study, the needle oils of *P. nigra*, *P. peuce* and *P. heldreichii* and their fractions were evaluated for their DPPH radical scavenging activity, which was proven rather weak [32]. Similarly, *P. heldreichii* var. *leucodermis* needle oil from central Herzegovina exhibited weak DPPH radical scavenging activity [38]. The red pine needle oil (*P. densiflora*) has exerted a rather weak DPPH radical scavenging potential, as well as nitrite radical scavenging ability [34]. The needle oil of the maritime pine (*P. pinaster*) has been evaluated by Tümen et al. for its antioxidant potential using the DPPH, ABTS/TEAC and FRAP assays, as well as for its hydroxyl radical scavenging activity, displaying a rather moderate potential [39]. The Monterey pine (*P. radiata*) needle oil, evaluated for its antioxidant capacity using the DPPH, BCB and LCL assays, exhibited a rather moderate to weak activity in all three tests [40], similarly to our results. The Japanese white pine (*P. parviflora*) needle oil has demonstrated weaker DPPH scavenging activity compared to thymol, but strong hydroxyl radical scavenging activity in reference to mannitol [27]. *P. massoniana* needle oil has exerted low to moderate antioxidant potential, as determined using the DPPH, ABTS/TEAC and FRAP assays [35], while the Chir pine (*P. roxburghii*) needle oil has showed weak DPPH radical scavenging activity [41].

To the best of our knowledge, this is the first report on the evaluation of the antioxidant potential of the essential oils of *P. canariensis*, *P. mugo* var. *prostrata*, *P. mugo* var. *pumilio*, *P. nigra* var. *caramanica*, *P. nigra* var. *laricio*, *P. nigra* subsp. *nigra*, *P. nigra* var. *salzmanii*, *P. sylvestris* subsp. *scotica*, *P. taiwanensis*, *P. attenuata, P. elliottii, P. muricata, P. patula, P. rigida, P. teocote, P. banksiana, P. contorta* var. *contorta*, *P. contorta* var. *latifolia*, *P. contorta* var. *murrayana, P. coulteri, P. jeffreyi, P. ponderosa, P. sabineana, P. torreyana, P. aristata*, *P. cembroides*, *P. culminicola*, *P. monophylla*, *P. bungeana*, *P. gerardiana*, *P. armandii*, *P. flexilis*, *P. koraiensis*, *P. monticola*, *P. pumila*, *P. strobiformis*, and *P. strobus*.

### 3.2. Evaluation of the Antioxidant Activity of Extracts

In the framework of the present study, two extracts of different polarity, namely an organic extract resulting from maceration of the needles in CH_2_Cl_2_/EtOH (2:1) containing less polar constituents and a hydroethanolic extract resulting from maceration of the needles in EtOH/H_2_O (1:2) containing more polar constituents, were prepared from the fresh needles of 54 pine taxa and evaluated for their antioxidant potential using the LCL assay.

An overall comparison of the IC_50_ values of the investigated organic extracts (Table 1, Figure 1b) revealed the superiority of *P. contorta* var. *murrayana* of section *Trifoliae* (subgenus *Pinus*), followed by *P. nigra* subsp. *caramanica* and *P. nigra* subsp. *salzmanii* of section *Pinus* (subgenus *Pinus*), along with *P. monticola* of section *Quinquefoliae* (subgenus *Strobus*), with the organic extracts of the four taxa exhibiting stronger antioxidant activity than quercetin.

The antioxidant activity evaluation of the hydroethanolic extracts (Table 1, Figure 1c) showed that only *P. nigra* subsp. *nigra* exhibited a lower IC_50_ value than quercetin. However, significant levels of activity were also observed for the hydroethanolic extracts of *P. brutia*, *P. canariensis*, *P. tabuliformis*, *P. contorta* var. *latifolia*, *P. mugo* var. *pumilio*, *P. pinaster*, and *P. ponderosa*. All aforementioned taxa belong to the subgenus *Pinus*.

A number of studies employing different assays for the evaluation of the antioxidant activity of various pine needle extracts have been undertaken and their results are summarized in Table 2. Nonetheless, due to the different extraction protocols used in these investigations, direct comparison of the results obtained in the current study is not straightforward.

### 3.3. Phytochemical Analysis of P. nigra subsp. nigra and Evaluation of the Antioxidant Activity of the Isolated Metabolites

In the current study, both organic and hydroethanolic extracts, as well as the essential oil of the black pine (*P. nigra* subsp. *nigra*), were constantly among the most active samples tested, with IC_50_ values of 0.17 ± 0.01, 0.14 ± 0.02, and 2.05 ± 0.20, respectively. Therefore, phytochemical analysis of the black pine needle extract was undertaken, aiming at the isolation of the metabolites responsible for the observed antioxidant activity.

**Figure 2:**
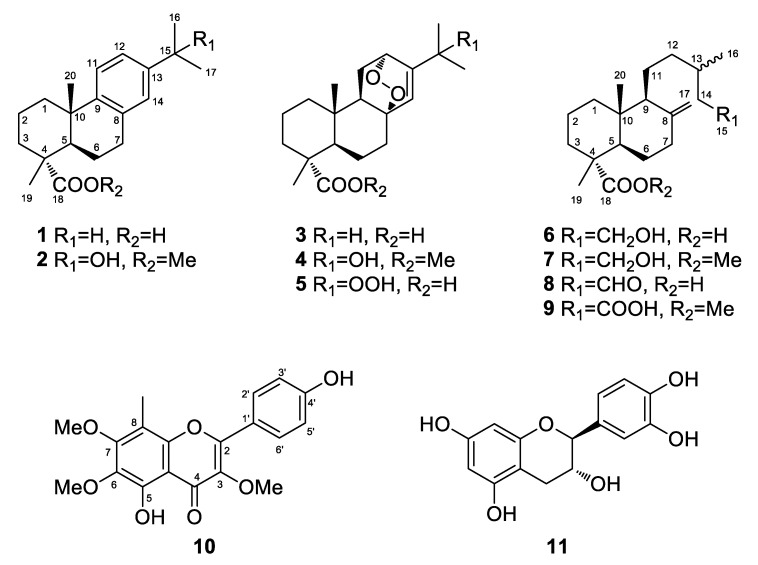
Structures of compounds **1**–**11** isolated from the organic extract of the fresh needles of *P. nigra* subsp. *nigra*.

A series of chromatographic separations of the organic extract of the fresh needles of *P. nigra* subsp. *nigra* led to the isolation of compounds **1**−**11** (Figure 2), which were identified as dehydroabietic acid (**1**) [50], 15-hydroxy-dehydroabietic methyl ester (**2**) [50], 8,12*α*-epidioxy-abiet-13-en-18-oic acid (**3**) [50], 15-hydroxy-8,12*α*-epidioxy-abiet-13-en-18-oic methyl ester (**4**) [50], 15-hydroperoxy-8,12*α*-epidioxy-abiet-13-en-18-oic acid (**5**) [50], 15-hydroxy-8(17)-labden-18-oic acid (**6**) [51], 15-hydroxy-8(17)-labden-18-oic methyl ester (**7**) [51], 15-oxo-8(17)-labden-18-oic acid (**8**) [51], 8(17)-labden-15,18-dioic acid 18-methyl ester (**9**) [51], 5,4′-dihydroxy-3,6,7-trimethoxy-8-*C*-methylflavone (**10**) [51,52], (-)-catechin (**11**) [51], a rare stereoisomer of catechin, and *β*-sitosterol [51] by comparison of their spectroscopic and physical characteristics with those reported in the literature. Among them, compounds **2**–**4**, **6**, **11** and *β*-sitosterol are reported for the first time from black pine, whereas metabolite **10** is reported for the first time in Gymnospermae. It is worth noting that the chemical structure of **10**, which has been reported from the leaves of three *Vellozia* species and the fungus *Colletotrichum dematium* f.sp. *epilobii*, has been so far only tentatively assigned [52,53].

The structure of compound **10** was elucidated after thorough analysis of its spectroscopic data. Specifically, according to the NMR and MS spectra, metabolite **10** was identified as a flavonol with a *para*-substituted ring B, bearing one aromatic methyl, two hydroxy and three methoxy groups. The positions of the functional groups were determined after analysis of a standard set of six UV spectra [54]. In particular, in the presence of NaOMe, band Ib exhibited a bathochromic shift of 56 nm with no decrease in intensity, typical of the presence of a free hydroxy group at C-4′. Moreover, no small additional peak or shoulder at 330 nm was observed, indicating the absence of a free hydroxy group at C-7. With AlCl_3_ and AlCl_3_-HCl, bathochromic shifts of 25 nm and 24 nm, respectively, were observed, diagnostic for the presence of 5-OH and 6-OMe in 3-*O*-substituted flavonols. No shift was observed in band II in the presence of NaOAc, verifying the presence of 6-OMe, as well as of a methyl group at C-8, also confirming a 7-*O*-substitution. The presence of 7-OMe was confirmed by the fact that no shift was observed in the presence of NaOAc and H_3_BO_3_. The proposed structure was further supported by the heteronuclear correlations observed in the HMBC spectrum of metabolite **10**. The ^1^H and ^13^C NMR chemical shifts for compound **10** are reported herein for the first time, complementing the relevant literature.

Metabolites **1**–**11** were subjected to evaluation of their antioxidant potential using the LCL assay (Table 3). Phenolic compounds **10** and **11** displayed significant levels of activity with IC_50_ values of 1.95 ± 0.21 and 1.34 ± 0.16 μg/mL, respectively, whereas the isolated diterpenes showed moderate levels of activity (**1** and **3**) or were proven inactive (**2** and **4**–**9**). The fact that both extracts of the black pine needles showed higher antioxidant activity compared to that of the isolated compounds indicates that the higher antioxidant potential of the extracts may be the result of synergism.

## 4. Conclusions

The antioxidant activity of the essential oils, as well as of the organic (CH_2_Cl_2_/EtOH 2:1) and hydroethanolic (EtOH/H_2_O 1:2) extracts of the fresh needles, from 54 pine taxa was evaluated using the POCL and LCL assays. The extracts showed overall higher I_0_ inhibition in comparison to the essential oils. Two samples from subgenus *Pinus* were proven to be the most potent among the investigated essential oils, namely *P. canariensis* (section *Pinus*) followed by *P. attenuata* oil (section *Trifoliae*), albeit with observed IC_50_ values higher than that of the reference (*β*-carotene). The organic extracts of *P. contorta* var. *murrayana* (section *Trifoliae*), followed by *P. nigra* subsp. *caramanica* (section *Pinus*), *P. nigra* subsp. *salzmanii* (section *Pinus*), *P. monticola* (section *Quinquefoliae*), *P. mugo* var. *prostrata* (section *Pinus*) and *P. sylvestris* subsp. *scotica* (section *Pinus*), exhibited the same or higher levels of activity compared to the reference (quercetin). Among the hydroethanolic extracts, however, only *P. nigra* subsp. *nigra* (section *Pinus*) demonstrated stronger antioxidant activity than that of the reference (quercetin), albeit with several other taxa of subgenus *Pinus* displaying significant levels of activity.

Based on the overall levels of activity, *P. nigra* subsp. *nigra* was selected for phytochemical analysis targeting the isolation of the bioactive constituents. Among the secondary metabolites isolated from the organic extract of the black pine needles, the abietane and labdane diterpenes **1**–**9** were not active, whereas the two phenolic compounds **10** and **11** showed noteworthy levels of antioxidant activity. To the best of our knowledge, this is the first report on the evaluation of the antioxidant activity of the needle essential oils and extracts from 37 and 41 pine taxa, respectively.

## Figures and Tables

**Table 1:** Antioxidant activity (expressed as IC_50_ in μg/mL) of the essential oils, the organic (CH_2_Cl_2_/EtOH 2:1) and the hydroethanolic (EtOH/H_2_O 1:2) extracts of the fresh needles of 54 *Pinus* taxa.

Taxon				IC_50_ (μg/mL)			
				Essential Oil	Organic Extract		Hydroethanolic Extract
Subgenus *Pinus*							
	Section *Pinus*						
		Subsection *Pinaster*					
1			*P. brutia*	4.67 ± 0.14	0.27 ± 0.01		0.20 ± 0.02
2			*P. canariensis*	1.00 ± 0.08	0.29 ± 0.05		0.20 ± 0.01
3			*P. halepensis*	1.78 ± 0.17	0.30 ± 0.03		0.90 ± 0.02
4			*P. heldreichii*	7.26 ± 0.54	0.33 ± 0.02		0.66 ± 0.08
5			*P. pinaster*	7.03±1.12	0.21 ± 0.01		0.25 ± 0.02
6			*P. pinea*	4.40 ± 0.37	0.30 ± 0.03		0.40 ± 0.02
7			*P. roxburghii*	15.96±1.45	0.30 ± 0.01		0.34 ± 0.01
		Subsection *Pinus*					
8			*P. densiflora*	4.45 ± 0.40	0.17 ± 0.01		0.28 ± 0.01
9			*P. massoniana*	8.29 ± 0.41	0.20 ± 0.02		0.41 ± 0.02
10			*P. mugo*	1.89 ± 0.16	0.19 ± 0.01		0.34 ± 0.01
11			*P. mugo* var. *prostrata*	1.79 ± 0.21	0.15 ± 0.01		0.33 ± 0.01
12			*P. mugo* var. *pumilio*	3.42 ± 0.06	0.27 ± 0.01		0.24 ± 0.01
13			*P. nigra* subsp. *caramanica*	3.28 ± 0.27	0.08 ± 0.01		0.27 ± 0.01
14			*P. nigra* subsp. *laricio*	5.25 ± 0.19	0.30 ± 0.02		0.35 ± 0.02
15			*P. nigra* subsp. *nigra*	2.05 ± 0.20	0.17 ± 0.01		0.14 ± 0.02
16			*P. nigra* subsp. *salzmannii*	4.05 ± 0.12	0.12 ± 0.03		0.66 ± 0.03
17			*P. sylvestris*	4.86 ± 0.48	0.17 ± 0.04		0.37 ± 0.01
18			*P. sylvestris* subsp. *scotica*	1.67 ± 0.05	0.15 ± 0.01		0.30 ± 0.01
19			*P. tabuliformis*	3.97 ± 0.62	0.30 ± 0.01		0.22 ± 0.04
20			*P. taiwanensis*	9.31 ± 0.29	0.19 ± 0.02		0.42 ± 0.02
21			*P. thunbergii*	2.68 ± 0.12	0.38 ± 0.04		0.41 ± 0.02
	Section *Trifoliae*						
		Subsection *Australes*					
22			*P. attenuata*	1.30 ± 0.02	0.20 ± 0.01		0.28 ± 0.02
23			*P. elliottii*	3.97 ± 0.35	0.32 ± 0.01		0.52 ± 0.03
24			*P. muricata*	1.60 ± 0.09	0.24 ± 0.01		0.40 ± 0.03
25			*P. patula*	5.63 ± 0.02	0.36 ± 0.02		0.43 ± 0.02
26			*P. radiata*	5.65 ± 0.10	0.35 ± 0.04		0.41 ± 0.03
27			*P. rigida*	2.09 ± 0.12	0.16 ± 0.01		0.59 ± 0.02
28			*P. teocote*	5.36±1.23	0.29 ± 0.03		0.56 ± 0.06
		Subsection *Contortae*					
29			*P. banksiana*	3.60 ± 0.14	0.31 ± 0.03		0.44 ± 0.02
30			*P. contorta* var. *contorta*	5.11 ± 0.40	0.22 ± 0.00		1.30 ± 0.10
31			*P. contorta* var. *latifolia*	9.57 ± 0.64	0.31 ± 0.02		0.22 ± 0.01
32			*P. contorta* var. *murrayana*	3.51 ± 0.16	0.06 ± 0.00		0.41 ± 0.02
		Subsection *Ponderosae*					
33			*P. coulteri*	2.64 ± 0.08	0.24 ± 0.01		0.35 ± 0.01
34			*P. jeffreyi*	3.72 ± 0.39	0.20 ± 0.00		0.57 ± 0.03
35			*P. ponderosa*	2.86 ± 0.09	0.19 ± 0.01		0.25 ± 0.01
36			*P. sabineana*	9.05±1.25	0.31 ± 0.04		0.36 ± 0.05
37			*P. torreyana*	9.58 ± 0.40	0.28 ± 0.01		0.37 ± 0.03
Subgenus *Strobus*							
	Section *Parrya*						
		Subsection *Balfourianae*					
38			*P. aristata*	16.39±1.52	0.27 ± 0.01		0.28 ± 0.02
		Subsection *Cembroides*					
39			*P. cembroides*	2.38 ± 0.16	0.41 ± 0.02		1.43 ± 0.11
40			*P. culminicola*	11.71±2.17	0.17 ± 0.01		1.00 ± 0.05
41			*P. monophylla*	20.03±2.77	0.39 ± 0.01		0.66 ± 0.04
	Section *Quinquefoliae*						
		Subsection *Gerardianae*					
42			*P. bungeana*	4.99 ± 0.14	0.28 ± 0.02		0.40 ± 0.02
43			*P. gerardiana*	11.35±2.03	0.44 ± 0.05		0.46 ± 0.01
		Subsection *Strobus*					
44			*P. armandii*	4.95 ± 0.17	0.58 ± 0.03		0.48 ± 0.07
45			*P. cembra*	2.36 ± 0.05	0.40 ± 0.02		0.67 ± 0.02
46			*P. flexilis*	3.62 ± 0.57	0.27 ± 0.01		0.36 ± 0.01
47			*P. koraiensis*	2.73 ± 0.07	0.25 ± 0.02		0.55 ± 0.03
48			*P. monticola*	1.94 ± 0.09	0.14 ± 0.03		0.45 ± 0.02
49			*P. parviflora*	7.04 ± 0.44	1.34 ± 0.01		0.38 ± 0.03
50			*P. peuce*	4.04 ± 0.26	0.35 ± 0.01		0.67 ± 0.03
51			*P. pumila*	4.24 ± 0.27	0.19 ± 0.01		0.75 ± 0.06
52			*P. strobiformis*	2.68 ± 0.15	0.16 ± 0.02		0.47 ± 0.10
53			*P. strobus*	11.54±3.27	0.22 ± 0.04		0.35 ± 0.02
54			*P. wallichiana*	2.23 ± 0.12	0.43 ± 0.04		0.49 ± 0.04
			*β*-carotene	0.23 ± 0.01			
			quercetin			0.15 ± 0.00	

**Table 2:** Antioxidant activity of various needle extracts of *Pinus* taxa previously reported in the literature.

Species	Extract	Assay	Activity	Reference
*P. brutia*	Fresh needles, CHCl_3_/MeOH (3:1) extract, organic phase F1	LCL ^1^	165.8 ± 0.06 μg/mL ^9^	[42]
	Fresh needles, CHCl_3_/MeOH (3:1) extract, organic phase F2	LCL ^1^	31.89 ± 0.02 μg/mL ^9^	
	Dry needles, CHCl_3_/MeOH (3:1) extract, organic phase F1	LCL ^1^	327.5 ± 0.08 μg/mL ^9^	
	Dry needles, CHCl_3_/MeOH (3:1) extract, organic phase F2	LCL ^1^	18.38 ± 0.06 μg/mL ^9^	
	Dry needles, Me_2_CO extract	DPPH ^2^	10.36 ± 0.13–16.00 ± 0.26% (at 250–1000 μg/mL) ^10^	[37]
		DMPD ^3^	inactive (at 250–1000 μg/mL) ^10^	
		PFRAP ^4^	0.316 ± 0.042–0.889 ± 0.011 (at 250–1000 μg/mL) ^11^	
	Dry needles, EtOAc extract	DPPH ^2^	14.14 ± 0.45–28.27 ± 0.26% (at 250–1000 μg/mL) ^10^	
		DMPD ^3^	2.15 ± 0.56–12.66 ± 2.14% (at 250–1000 μg/mL) ^10^	
		PFRAP ^4^	0.311 ± 0.013–0.792 ± 0.033 (at 250–1000 μg/mL) ^11^	
	Dry needles, EtOH extract	DPPH ^2^	13.41 ± 0.19–25.59 ± 0.19% (at 250–1000 μg/mL) ^10^	
		DMPD ^3^	inactive–7.72 ± 1.24% (at 250–1000 μg/mL) ^10^	
		PFRAP ^4^	0.229 ± 0.042–0.630 ± 0.037 (at 250–1000 μg/mL) ^11^	
	Dry needles, MeOH extract	DPPH ^2^	27.5 ± 0.4–85.0 ± 0.8% (at 0.2-1.0 mg/mL) ^10^	[36]
		PFRAP ^4^	0.119 ± 0.009–0.438 ± 0.008 (at 0.2–0.8 mg/mL) ^11^	
		FICA ^5^	21.5 ± 0.4% (at 1.0 mg/mL) ^12^	
*P. halepensis*	Fresh needles, CHCl_3_/MeOH (3:1) extract, organic phase F1	LCL ^1^	175.0 ± 0.03 μg/mL ^9^	[42]
	Fresh needles, CHCl_3_/MeOH (3:1) extract, organic phase F2	LCL ^1^	inactive	
	Dry needles, Me_2_CO extract	DPPH ^2^	7.61 ± 0.20–31.18 ± 1.02% (at 250–1000 μg/mL) ^10^	[37]
		DMPD ^3^	13.32 ± 0.98–17.66 ± 1.65% (at 250–1000 μg/mL) ^10^	
		PFRAP ^4^	0.330 ± 0.008–0.941 ± 0.018 (at 250–1000 μg/mL) ^11^	
	Dry needles, EtOAc extract	DPPH ^2^	inactive–21.05 ± 0.71% (at 250–1000 μg/mL) ^10^	
		DMPD ^3^	5.43 ± 1.44–9.96 ± 0.57% (at 250–1000 μg/mL) ^10^	
		PFRAP ^4^	0.264 ± 0.012–0.849 ± 0.010 (at 250–1000 μg/mL) ^11^	
	Dry needles, EtOH extract	DPPH ^2^	8.98 ± 0.79–18.39 ± 1.22% (at 250–1000 μg/mL) ^10^	
		DMPD ^3^	inactive (at 250–1000 μg/mL) ^10^	
		PFRAP ^4^	0.412 ± 0.042–1.250 ± 0.022 (at 250–1000 μg/mL) ^11^	
	Dry needles, MeOH extract	DPPH ^2^	43.1 ± 3.1–93.9 ± 0.1% (at 0.2-1.0 mg/mL) ^10^	[36]
		PFRAP ^4^	0.236 ± 0.010–0.914 ± 0.008 (at 0.2–0.8 mg/mL) ^11^	
		FICA ^5^	5.5 ± 0.8% (at 1.0 mg/mL) ^12^	
*P. pinaster*	Dry needles, Me_2_CO (80%) extract, filtrate	ORAC ^6^	478.8 ± 32.8 μM TE/g ^13^	[43]
	Dry needles, Me_2_CO (80%) extract, alkaline hydrolysis of the residue/EtOAc-soluble fraction	ORAC ^6^	128.0 ± 9.6 μM TE/g ^13^	
	Dry needles, Me_2_CO (80%) extract, alkaline hydrolysis of the residue/H_2_O-soluble fraction	ORAC ^6^	60.2±7.1 μM TE/g ^13^	
	Fresh needles, *n*-Hex extract	DPPH ^2^	203.28 μg/mL ^9^	[39]
		ABTS/TEAC ^7^	170.92 μg/mL ^9^	
		FRAP ^8^	16.28% (concentration not specified) ^14^	
		hydroxyl radical scavenging	158.26 μg/mL ^9^	
	Fresh needles, Me_2_CO extract (sequentially)	DPPH ^2^	171.12 μg/mL ^9^	
		ABTS/TEAC ^7^	163.45 μg/mL ^9^	
		FRAP ^8^	19.74% (concentration not specified) ^14^	
		hydroxyl radical scavenging	192.35 μg/mL ^9^	
*P. pinea*	Fresh needles, CHCl_3_/MeOH (3:1) extract, organic phase F1	LCL ^1^	161.8 ± 0.07 μg/mL ^9^	[42]
	Fresh needles, CHCl_3_/MeOH (3:1) extract, organic phase F2	LCL ^1^	129.6 ± 0.04 μg/mL ^9^	
	Dry needles, CHCl_3_/MeOH (3:1) extract, organic phase F1	LCL ^1^	42.1 ± 0.01 μg/mL ^9^	
	Dry needles, CHCl_3_/MeOH (3:1) extract, organic phase F2	LCL ^1^	79.2 ± 0.03 μg/mL ^9^	
	Dry needles, Me_2_CO (80%) extract, filtrate	ORAC ^6^	901.5 ± 35.2 μM TE/g ^13^	[43]
	Dry needles, Me_2_CO (80%) extract, alkaline hydrolysis of the residue/EtOAc-soluble fraction	ORAC ^6^	70.9 ± 0.9 μM TE/g ^13^	
	Dry needles, Me_2_CO (80%) extract, alkaline hydrolysis of the residue/H_2_O-soluble fraction	ORAC ^6^	39.7±5.5 μM TE/g ^13^	
	Dry needles, MeOH extract	DPPH ^2^	27.9 ± 0.8–91.4 ± 0.5% (at 0.2-1.0 mg/mL) ^10^	[36]
		PFRAP ^4^	0.154 ± 0.016–0.542 ± 0.031 (at 0.2–0.8 mg/mL) ^11^	
		FICA ^5^	1.2 ± 0.4% (at 1.0 mg/mL) ^12^	
*P. roxburghii*	Dry needles, *n*-Hex fraction of MeOH extract	DPPH ^2^	inactive	[44]
	Dry needles, CH_2_Cl_2_ fraction of MeOH extract	DPPH ^2^	163.45 μg/mL ^9^	
	Dry needles, EtOAc fraction of MeOH extract	DPPH ^2^	11.62 μg/mL ^9^	
	Dry needles, *n*-BuOH fraction of MeOH extract	DPPH ^2^	3.283 μg/mL ^9^	
	Dry needles, H_2_O fraction of MeOH extract	DPPH ^2^	120.0 μg/mL ^9^	
	Dry needles, EtOH (95%) extract	ABTS/TEAC ^7^	0.57 mM (maximum TEAC content at 12.5 μg/mL)	[45]
	Dry needles, *n*-Hex fraction of EtOH (95%) extract	ABTS/TEAC ^7^	inactive	
	Dry needles, CHCl_3_ fraction of EtOH (95%) extract	ABTS/TEAC ^7^	0.14 mM (maximum TEAC content at 12.5 μg/mL)	
	Dry needles, *n*-BuOH fraction of EtOH (95%) extract	ABTS/TEAC ^7^	0.38 mM (maximum TEAC content at 12.5 μg/mL)	
	Dry needles, *n*-BuOH-insoluble fraction of EtOH (95%) extract	ABTS/TEAC ^7^	0.57 mM (maximum TEAC content at 12.5 μg/mL)	
*P. densiflora*	Dry needles, MeOH extract	DPPH ^2^	32.5 μg/mL ^9^	[46]
		nitrite radical scavenging	80.38 ± 1.44% (at 10 μg/mL) ^10^	
		hydroxyl radical scavenging	−29.79 ± 5.18% (at 40 μg/mL) ^10^	
		reactive oxygen species (ROS) scavenging	−392.80 ± 21.3% (at 40 μg/mL) ^10^	
	Dry needles, CH_2_Cl_2_ fraction of MeOH extract	DPPH ^2^	45.4 μg/mL ^9^	
		nitrite radical scavenging	21.36 ± 1.04% (at 10 μg/mL) ^10^	
		hydroxyl radical scavenging	−357.45 ± 10.4% (at 40 μg/mL) ^10^	
		reactive oxygen species (ROS) scavenging	−907.36 ± 50.0% (at 40 μg/mL) ^10^	
	Dry needles, EtOAc fraction of MeOH extract	DPPH ^2^	13.2 μg/mL ^9^	
		nitrite radical scavenging	95.60 ± 0.09% (at 10 μg/mL) ^10^	
		hydroxyl radical scavenging	82.13 ± 5.31% (at 40 μg/mL) ^10^	
		reactive oxygen species (ROS) scavenging	59.15 ± 3.4% (at 40 μg/mL) ^10^	
	Dry needles, *n*-BuOH fraction of MeOH extract	DPPH ^2^	24.3 μg/mL ^9^	
		nitrite radical scavenging	82.28 ± 1.89% (at 10 μg/mL) ^10^	
		hydroxyl radical scavenging	61.70 ± 4.42% (at 40 μg/mL) ^10^	
		reactive oxygen species (ROS) scavenging	50.55 ± 3.7% (at 40 μg/mL) ^10^	
	Dry needles, H_2_O fraction of MeOH extract	DPPH ^2^	25.1 μg/mL ^9^	
		nitrite radical scavenging	69.02 ± 1.29% (at 10 μg/mL) ^10^	
		hydroxyl radical scavenging	27.66 ± 0.43% (at 40 μg/mL) ^10^	
		reactive oxygen species (ROS) scavenging	40.38 ±3.20% (at 40 μg/mL) ^10^	
	Dry needles, Me_2_CO (80%) extract, filtrate	ORAC ^6^	466.1±27.3 μM TE/g ^13^	[43]
	Dry needles, Me_2_CO (80%) extract, alkaline hydrolysis of the residue/EtOAc-soluble fraction	ORAC ^6^	61.4±3.7 μM TE/g ^13^	
	Dry needles, Me_2_CO (80%) extract, alkaline hydrolysis of the residue/H_2_O-soluble fraction	ORAC ^6^	55.3±2.8 μM TE/g ^13^	
	Dry needles, EtOH (95%) extract	inhibition of lipid peroxidation	53.48 μg/mL ^9^	[47]
		DPPH ^2^	95.12 μg/mL ^9^	
	Dry needles, H_2_O extract	DPPH ^2^	176.37±29.84 μg/mL ^9^	[48]
		ABTS/TEAC ^7^	14.90 ± 0.37 μg/mL ^9^	
	Dry needles, EtOH (20%) extract	DPPH ^2^	83.70 ± 6.22 μg/mL ^9^	
		ABTS/TEAC ^7^	9.02 ± 0.55 μg/mL ^9^	
	Dry needles, EtOH (40%) extract	DPPH ^2^	75.96 ± 11.60 μg/mL ^9^	
		ABTS/TEAC ^7^	8.56 ± 0.51 μg/mL ^9^	
	Dry needles, EtOH (60%) extract	DPPH ^2^	78.46 ± 7.99 μg/mL ^9^	
		ABTS/TEAC ^7^	9.12 ± 0.43 μg/mL ^9^	
	Dry needles, EtOH (80%) extract	DPPH ^2^	126.47 ± 4.38 μg/mL ^9^	
		ABTS/TEAC ^7^	11.80 ± 0.08 μg/mL ^9^	
	Dry needles, EtOH (100%) extract	DPPH ^2^	373.70 ± 60.67 μg/mL ^9^	
		ABTS/TEAC ^7^	19.76 ± 1.32 μg/mL ^9^	
*P. nigra*	Fresh needles, CHCl_3_/MeOH (3:1) extract, organic phase F1	LCL ^1^	inactive	[42]
	Fresh needles, CHCl_3_/MeOH (3:1) extract, organic phase F2	LCL ^1^	174.6 ± 0.15 μg/mL ^9^	
	Dry needles, Me_2_CO extract	DPPH ^2^	10.14 ± 0.58–17.14 ± 1.09% (at 250–1000 μg/mL) ^10^	[37]
		DMPD ^3^	inactive (at 250–1000 μg/mL) ^10^	
		PFRAP ^4^	0.273 ± 0.022–0.893 ± 0.078 (at 250–1000 μg/mL) ^11^	
	Dry needles, EtOAc extract	DPPH ^2^	12.91 ± 0.26–24.36 ± 1.80% (at 250–1000 μg/mL) ^10^	
		DMPD ^3^	inactive (at 250–1000 μg/mL) ^10^	
		PFRAP ^4^	0.346 ± 0.001–0.969 ± 0.041 (at 250–1000 μg/mL) ^11^	
	Dry needles, EtOH extract	DPPH ^2^	14.41 ± 1.09–28.36 ± 0.77% (at 250–1000 μg/mL) ^10^	
		DMPD ^3^	inactive (at 250–1000 μg/mL) ^10^	
		PFRAP ^4^	0.360 ± 0.024–0.965 ± 0.029 (at 250–1000 μg/mL) ^11^	
	Dry needles, MeOH extract	DPPH ^2^	34.0 ± 2.1–92.5 ± 0.4% (at 0.2-1.0 mg/mL) ^10^	[36]
		PFRAP ^4^	0.163 ± 0.002–0.586 ± 0.008 (at 0.2–0.8 mg/mL) ^11^	
		FICA ^5^	21.3±2.1% (at 1.0 mg/mL) ^12^	
*P. sylvestris*	Dry needles, Me_2_CO (80%) extract, filtrate	ORAC ^6^	560.0±36.3 μM TE/g ^13^	[43]
	Dry needles, Me_2_CO (80%) extract, alkaline hydrolysis of the residue/EtOAc-soluble fraction	ORAC ^6^	91.7±3.2 μM TE/g ^13^	
	Dry needles, Me_2_CO (80%) extract, alkaline hydrolysis of the residue/H_2_O-soluble fraction	ORAC ^6^	59.3±4.0 μM TE/g ^13^	
	Dry needles, Me_2_CO extract	DPPH ^2^	15.77 ± 1.74–31.41 ± 0.84% (at 250–1000 μg/mL) ^10^	[37]
		DMPD ^3^	inactive–4.22 ± 0.11 (at 250–1000 μg/mL) ^10^	
		PFRAP ^4^	0.327 ± 0.048–1.015 ± 0.066 (at 250–1000 μg/mL) ^11^	
	Dry needles, EtOAc extract	DPPH ^2^	8.32 ± 0.19–13.55 ± 0.01% (at 250–1000 μg/mL) ^10^	
		DMPD ^3^	inactive (at 250–1000 μg/mL) ^10^	
		PFRAP ^4^	0.230 ± 0.013–0.627 ± 0.011 (at 250–1000 μg/mL) ^11^	
	Dry needles, EtOH extract	DPPH ^2^	22.64±1.41–45.86±1.35% (at 250–1000 μg/mL) ^10^	
		DMPD ^3^	3.03 ± 0.45–14.57 ± 1.91% (at 250–1000 μg/mL) ^10^	
		PFRAP ^4^	0.515 ± 0.005–1.343 ± 0.013 (at 250–1000 μg/mL) ^11^	
*P. attenuata*	Fresh needles, CHCl_3_/MeOH (3:1) extract, organic phase F1	LCL ^1^	inactive	[42]
	Fresh needles, CHCl_3_/MeOH (3:1) extract, organic phase F2	LCL ^1^	144.1 ± 0.01 μg/mL ^9^	
*P. radiata*	Fresh needles, CHCl_3_/MeOH (3:1) extract, organic phase F1	LCL ^1^	228.1 ± 0.02 μg/mL ^9^	[42]
	Fresh needles, CHCl_3_/MeOH (3:1) extract, organic phase F2	LCL ^1^	inactive	
*P. cembra*	Dry needles, MeOH (80%) extract	DPPH ^2^	186.1 ± 1.7 μg/mL ^9^	[49]
		ABTS/TEAC ^7^	24.0 ± 0.2 μg/mL ^9^	
		PFRAP ^4^	104 ± 2 μg/mL ^9^	
		FICA ^5^	1755 ± 22 μg/mL ^9^	
*P. koraiensis*	Dry needles, Me_2_CO (80%) extract, filtrate	ORAC ^6^	402.0±7.5 μM TE/g ^13^	[43]
	Dry needles, Me_2_CO (80%) extract, alkaline hydrolysis of the residue/EtOAc-soluble fraction	ORAC ^6^	111.6±6.2 μM TE/g ^13^	
	Dry needles, Me_2_CO (80%) extract, alkaline hydrolysis of the residue/H_2_O-soluble fraction	ORAC ^6^	32.0±4.5 μM TE/g ^13^	
*P. strobus*	Dry needles, Me_2_CO (80%) extract, filtrate	ORAC ^6^	1223.3±12.6 μM TE/g ^13^	[43]
	Dry needles, Me_2_CO (80%) extract, alkaline hydrolysis of the residue/EtOAc-soluble fraction	ORAC ^6^	82.3±3.1 μM TE/g ^13^	
	Dry needles, Me_2_CO (80%) extract, alkaline hydrolysis of the residue/H_2_O-soluble fraction	ORAC ^6^	81.3±2.4 μM TE/g ^13^	
*P. wallichiana*	Dry needles, *n*-Hex fraction of MeOH extract	DPPH ^2^	inactive	[44]
	Dry needles, CH_2_Cl_2_ fraction of MeOH extract	DPPH ^2^	inactive	
	Dry needles, EtOAc fraction of MeOH extract	DPPH ^2^	8.403 μg/mL ^9^	
	Dry needles, *n*-BuOH fraction of MeOH extract	DPPH ^2^	85.90 μg/mL ^9^	
	Dry needles, H_2_O fraction of MeOH extract	DPPH ^2^	inactive	

^1^ luminol chemiluminescence, ^2^ 2,2-diphenyl-1-picrylhydrazyl radical scavenging, ^3^ *N*,*N*-dimethyl-*p*-phenylene diamine radical scavenging, ^4^ potassium ferricyanide reducing power, ^5^ iron (II) chelating ability employing the Fe^2+^-ferrozine system, ^6^ oxygen radical absorbance capacity, ^7^ 2,2-azino-bis-(3-ethylbenzothiazoline-6-sulphonate) radical cation scavenging or Trolox equivalent antioxidant capacity, ^8^ ferric reducing antioxidant power, ^9^ expressed as IC_50_ / EC_50_ in μg/mL, ^10^ expressed as % of scavenging activity (at a given concentration), ^11^ expressed as absorbance at 700 nm (at a given concentration), ^12^ expressed as % of chelating ability (at a given concentration), ^13^ expressed as μM Trolox equivalents (TE) per g dry weight, ^14^ expressed as % of reducing capacity (at a given concentration).

**Table 3:** Antioxidant activity (expressed as IC_50_ in μg/mL) of compounds **1**–**11** isolated from the organic extract of the fresh needles of *Pinus nigra* subsp. *nigra*.

Compound	IC_50_ (μg/mL)
**1**	35.52 ± 0.65
**2**	>100
**3**	25.91 ± 4.95
**4**	>100
**5**	92.45 ± 13.19
**6**	>100
**7**	>100
**8**	>100
**9**	>100
**10**	1.95 ± 0.21
**11**	1.34 ± 0.16

## References

[1] Augustyniak A., Bartosz G., Čipak A., Duburs G., Horáková L.U., Łuczaj W., Majekova M., Odysseos A.D., Rackova L., Skrzydlewska E. (2010). Natural and synthetic antioxidants: An updated overview. Free Radic. Res..

[2] Jennings B.H., Akoh C.C. (2009). Effectiveness of natural versus synthetic antioxidants in a rice bran oil-based structured lipid. Food Chem..

[3] Caleja C., Barros L., Antonio A.L., Oliveira M.B.P.P., Ferreira I.C.F.R. (2017). A comparative study between natural and synthetic antioxidants: Evaluation of their performance after incorporation into biscuits. Food Chem..

[4] Farjon A. (2005). Pines: Drawings and Description of the Genus Pinus.

[5] Gernandt D.S., Geada López G., Ortiz García S., Liston A. (2005). Phylogeny and classification of *Pinus*. Taxon.

[6] Packer L., Rimbach G., Virgili F. (1999). Antioxidant activity and biologic properties of a procyanidin-rich extract from pine (*Pinus maritima*) bark, Pycnogenol. Free Radic. Biol. Med..

[7] Ferreira-Santos P., Zanuso E., Genisheva Z., Rocha C.M.R., Teixeira J.A. (2020). Green and sustainable valorization of bioactive phenolic compounds from *Pinus* by-products. Molecules.

[8] Dridi W., Bordenave N. (2020). Pine bark phenolic extracts, current uses, and potential food applications: A review. Curr. Pharm. Des..

[9] Mármol I., Quero J., Jiménez-Moreno N., Rodríguez-Yoldi M.J., Ancín-Azpilicueta C. (2019). A systematic review of the potential uses of pine bark in food industry and health care. Trends Food Sci. Technol..

[10] El Omari N., Ezzahrae Guaouguaou F., El Menyiy N., Benali T., Aanniz T., Chamkhi I., Balahbib A., Taha D., Shariati M.A., Zengin G. (2020). Phytochemical and biological activities of *Pinus halepensis* mill., and their ethnomedicinal use. J. Ethnopharmacol..

[11] Jeong M.S., Park S.J., Han E.J., Park S.Y., Kim M.J., Jung K., Cho S.H., Kim S.Y., Yoon W.J., Ahn G. (2020). *Pinus thunbergii* PARL leaf protects against alcohol-induced liver disease by enhancing antioxidant defense mechanism in BALB/c mice. J. Funct. Foods.

[12] Chiu H.F., Wang H.M., Shen Y.C., Venkatakrishnan K., Wang C.K. (2019). Anti-inflammatory properties of fermented pine (*Pinus morrisonicola* Hay.) needle on lipopolysaccharide-induced inflammation in RAW 264.7 macrophage cells. J. Food Biochem..

[13] Lee J.S., Kim H.G., Lee H.W., Han J.M., Lee S.K., Kim D.W., Saravanakumar A., Son C.G. (2015). Hippocampal memory enhancing activity of pine needle extract against scopolamine-induced amnesia in a mouse model. Sci. Rep..

[14] Lee H., Kim H., Choue R., Lim H. (2016). Evaluation of the effects of *Pinus koraiensis* needle extracts on serum lipid and oxidative stress in adults with borderline dyslipidemia: A randomized, double-blind, and placebo-controlled clinical trial. Evid. Based Complement. Altern. Med..

[15] Yang H., Wang Z., Song W., Zhao Z., Zhao Y. (2021). Isolation of proanthocyanidins from *Pinus thunbergii* needles and tyrosinase inhibition activity. Process Biochem..

[16] EU Novel Food Catalogue. https://ec.europa.eu/food/safety/novel_food/catalogue_en.

[17] Sak K., Jürisoo K., Raal A. (2014). Estonian folk traditional experiences on natural anticancer remedies: From past to the future. Pharm. Biol..

[18] Kim K.-Y., Chung H.-J. (2000). Flavor compounds of pine sprout tea and pine needle tea. J. Agric. Food Chem..

[19] How to Make Pine Needle Tea. WikiHow. www.wikihow.com/Make-Pine-Needle-Tea.

[20] Pine Needle Recipes. Pinterest. www.pinterest.ca/arnica1281/pine-needle-recipes/.

[21] Ioannou E., Koutsaviti A., Tzakou O., Roussis V. (2014). The genus *Pinus*: A comparative study on the needle essential oil composition of 46 pine species. Phytochem. Rev..

[22] Arnous A., Petrakis C., Makris D., Kefalas P. (2002). A peroxyoxalate chemiluminescence-based assay for the evaluation of hydrogen peroxide scavenging activity employing 9,10-diphenylanthracene as the fluorophore. J. Pharmacol. Toxicol. Methods.

[23] Parejo I., Petrakis C., Kefalas P. (2000). A transition metal enhanced luminol chemiluminescence in the presence of a chelator. J. Pharmacol. Toxicol. Methods.

[24] Parejo I., Codina C., Petrakis C., Kefalas P. (2000). Evaluation of scavenging activity assessed by CoII/EDTA- induced chemiluminescence and DPPH (2,2-diphenyl-1-picrylhydrazyl) free radical assay. J. Pharmacol. Toxicol. Methods.

[25] Ruberto G., Baratta M.T. (2000). Antioxidant activity of selected essential oil components in two lipid model systems. Food Chem..

[26] Dar M.Y., Shah W.A., Mubashir S., Rather M.A. (2012). Chromatographic analysis, anti-proliferative and radical scavenging activity of *Pinus wallichiana* essential oil growing in high altitude areas of Kashmir, India. Phytomedicine.

[27] Chen J., Gao Y., Jin Y., Li S., Zhang Y. (2015). Chemical composition, antibacterial and antioxidant activities of the essential oil from needles of *Pinus parviflora* Siebold & Zucc. J. Essent. Oil Bear. Plants.

[28] Djerrad Z., Djouahri A., Kadik L. (2017). Variability of *Pinus halepensis* Mill. essential oils and their antioxidant activities depending on the stage of growth during vegetative cycle. Chem. Biodivers..

[29] Djerrad Z., Kadik L., Djouahri A. (2015). Chemical variability and antioxidant activities among *Pinushalepensis* Mill. essential oils provenances, depending on geographic variation and environmental conditions. Ind. Crops Prod..

[30] Postu P.A., Sadiki F.Z., El Idrissi M., Cioanca O., Trifan A., Hancianu M., Hritcu L. (2019). *Pinus halepensis* essential oil attenuates the toxic Alzheimer’s amyloid beta (1–42)-induced memory impairment and oxidative stress in the rat hippocampus. Biomed. Pharmacother..

[31] Grassmann J., Hippeli S., Vollmann R., Elstner E.F. (2003). Antioxidative properties of the essential oil from *Pinus mugo*. J. Agric. Food Chem..

[32] Kurti F., Giorgi A., Beretta G., Mustafa B., Gelmini F., Testa C., Angioletti S., Giupponi L., Zilio L.E., Pentimalli D. (2019). Chemical composition, antioxidant and antimicrobial activities of essential oils of different *Pinus* species from Kosovo. J. Essent. Oil Res..

[33] Apetrei C.L., Spac A., Brebu M., Tuchilus C., Miron A. (2013). Composition, and antioxidant and antimicrobial activities of the essential oils of a full-grown *Pinus cembra* L. tree from the Calimani Mountains (Romania). J. Serb. Chem. Soc..

[34] Park J.-S., Lee G.-H. (2011). Volatile compounds and antimicrobial and antioxidant activities of the essential oils of the needles of *Pinus densiflora* and *Pinus thunbergii*. J. Sci. Food Agric..

[35] Xie Q., Liu Z., Li Z. (2015). Chemical composition and antioxidant activity of essential oil of six *Pinus* taxa native to China. Molecules.

[36] Yener H.O., Saygideger S.D., Sarikurkcu C., Yumrutas O. (2014). Evaluation of antioxidant activities of essential oils and methanol extracts of *Pinus* species. J. Essent. Oil Bear. Plants.

[37] Ustun O., Senola F.S., Kurkcuoglu M., Orhan I.E., Kartal M., Baser K.H.C. (2012). Investigation on chemical composition, anticholinesterase and antioxidant activities of extracts and essential oils of Turkish *Pinus* species and pycnogenol. Ind. Crops Prod..

[38] Maric S., Jukic M., Katalinic V., Milos M. (2007). Comparison of chemical composition and free radical scavenging ability of glycosidically bound and free volatiles from Bosnian Pine (*Pinus heldreichii* Christ. var. *leucodermis*). Molecules.

[39] Tümen I., Akkol E.K., Taştan H., Süntar I., Kurtca M. (2018). Research on the antioxidant, wound healing, and anti-inflammatory activities and the phytochemical composition of maritime pine (*Pinus pinaster* Ait). J. Ethnopharmacol..

[40] Sacchetti G., Maietti S., Muzzoli M., Scaglianti M., Manfredini S., Radice M., Bruni R. (2005). Comparative evaluation of 11 essential oils of different origin as functional antioxidants, antiradicals and antimicrobials in foods. Food Chem..

[41] Salem M.Z.M., Ali H.M., Basalah M.O. (2014). Essential oils from wood, bark, and needles of *Pinus roxburghii* Sarg. from Alexandria, Egypt: Antibacterial and antioxidant activities. BioResources.

[42] Guri A., Kefalas P., Roussis V. (2006). Antioxidant potential of six pine species. Phytother. Res..

[43] Kang Y.-H., Howard L.R. (2010). Phenolic composition and antioxidant activities of different solvent extracts from pine needles in *Pinus* species. Food Sci. Nutr..

[44] Maimoona A., Naeem I., Shujaat S., Saddique Z., Mughal T., Mehmood T. (2011). Comparison of radical scavenging capacity of different extracts of barks and needles of *Pinus roxburghii* and *Pinus wallichiana*. Asian J. Chem..

[45] Puri A., Srivastava A.K., Singhal B., Mishra S.K., Srivastava S., Lakshmi V. (2011). Antidyslipidemic and antioxidant activity of *Pinus roxburghii* needles. Med. Chem. Res..

[46] Jung M.J., Chung H.Y., Choi J.H., Choi J.S. (2003). Antioxidant principles from the needles of red pine, *Pinus densiflora*. Phytother. Res..

[47] Kwak C.S., Moon S.C., Lee M.S. (2006). Antioxidant, antimutagenic, and antitumor effects of pine needles (*Pinus densiflora*). Nutr. Cancer.

[48] Venkatesan T., Choi Y.-W., Kim Y.-K. (2019). Effect of an extraction solvent on the antioxidant quality of *Pinus densiflora* needle extract. J. Pharm. Anal..

[49] Apetrei C.L., Tuchilus C., Aprotosoaie A.C., Oprea A., Malterud K.E., Miron A. (2011). Chemical, antioxidant and antimicrobial investigations of *Pinus cembra* L. bark and needles. Molecules.

[50] Koutsaviti A., Ioannou E., Couladis M., Tzakou O., Roussis V. (2017). ^1^H and ^13^C NMR spectral assignments of abietane diterpenes from *Pinus heldreichii* and *Pinus nigra* subsp. *nigra*. Magn. Reson. Chem..

[51] Koutsaviti A. (2016). Comparative Study of the Essential Oil Composition of the Foliage of 54 *Pinus* Taxa—Isolation and Structure Elucidation of Secondary Metabolites from the Species *Pinus heldreichii* Christ., *Pinus pinea* L. and *Pinus nigra* subsp. *nigra* Arn. Ph.D. Thesis.

[52] Abou-Zaid M., Dumas M., Chauret D., Watson A., Thompson D. (1997). C-Methyl flavonols from the fungus *Colletotrichum dematium* f. sp. *epilobii*. Phytochemistry.

[53] Harborne J.B., Greenham J., Williams C.A., Eagles J., Markham K.R. (1993). Ten isoprenylated and C-methylated flavonoids from the leaves of three *Vellozia* species. Phytochemistry.

[54] Mabry T.J., Markham K.R., Thomas M.B. (1970). The Systematic Identification of Flavonoids.

